# Well-Blended PCL/PEO Electrospun Nanofibers with Functional Properties Enhanced by Plasma Processing

**DOI:** 10.3390/polym12061403

**Published:** 2020-06-22

**Authors:** Vojtěch Kupka, Eva Dvořáková, Anton Manakhov, Miroslav Michlíček, Josef Petruš, Lucy Vojtová, Lenka Zajíčková

**Affiliations:** 1 Central European Institute of Technology—CEITEC, Brno University of Technology, Purkyňova 123, 61200 Brno, Czech Republic; vojtech.kupka@upol.cz (V.K.); josef.petrus@ceitec.vutbr.cz (J.P.); Lucy.Vojtova@ceitec.vutbr.cz (L.V.); 2 Regional Centre of Advanced Technologies and Materials and Department of Physical Chemistry, Faculty of Science, Palacký University in Olomouc, 17 Listopadu 12, 77900 Olomouc, Czech Republic; 3 Central European Institute of Technology—CEITEC, Masaryk University, Kamenice 5, 62500 Brno, Czech Republic; evke.dvorakova@gmail.com (E.D.); ant-manahov@ya.ru (A.M.); michlicekm@mail.muni.cz (M.M.); 4 Department of Physical Electronics, Faculty of Science, Masaryk University, Kotlářská 2, 61137 Brno, Czech Republic; 5 Laboratory of Inorganic Nanomaterials, National University of Science and Technology “MISiS”, Leninsky Prospect 4, 119049 Moscow, Russia; 6 Institute of Materials Chemistry, Faculty of Chemistry, Brno University of Technology, Purkyňova 464/118, 61200 Brno, Czech Republic

**Keywords:** polymer fibers, thin films, plasma enhanced CVD, mechanical properties, SEM

## Abstract

Biodegradable composite nanofibers were electrospun from poly(ε-caprolactone) (PCL) and poly(ethylene oxide) (PEO) mixtures dissolved in acetic and formic acids. The variation of PCL:PEO concentration in the polymer blend, from 5:95 to 75:25, revealed the tunability of the hydrolytic stability and mechanical properties of the nanofibrous mats. The degradation rate of PCL/PEO nanofibers can be increased compared to pure PCL, and the mechanical properties can be improved compared to pure PEO. Although PCL and PEO have been previously reported as immiscible, the electrospinning into nanofibers having restricted dimensions (250–450 nm) led to a microscopically mixed PCL/PEO blend. However, the hydrolytic stability and tensile tests revealed the segregation of PCL into few-nanometers-thin fibrils in the PEO matrix of each nanofiber. A synergy phenomenon of increased stiffness appeared for the high concentration of PCL in PCL/PEO nanofibrous mats. The pure PCL and PEO mats had a Young’s modulus of about 12 MPa, but the mats made of high concentration PCL in PCL/PEO solution exhibited 2.5-fold higher values. The increase in the PEO content led to faster degradation of mats in water and up to a 20-fold decrease in the nanofibers’ ductility. The surface of the PCL/PEO nanofibers was functionalized by an amine plasma polymer thin film that is known to increase the hydrophilicity and attach proteins efficiently to the surface. The combination of different PCL/PEO blends and amine plasma polymer coating enabled us to tune the surface functionality, the hydrolytic stability, and the mechanical properties of biodegradable nanofibrous mats.

## 1. Introduction

The artificial spinning of fibers is an essential part of current bioinspired technologies. It benefits from the progress in a variety of methods, including wet-spinning, dry-spinning, electrospinning, microfluidic spinning, direct drawing, and direct writing [1]. Electrospinning can be used to produce mats of single-component polymer fibers with sub-micrometer diameters or prepare different types of composites [2,3,4]. The mats provide a large specific surface area, high porosity, and good pore interconnectivity [5]. The morphology of electrospun mats is very similar to the structure of the natural extracellular matrix that supports cell attachment and proliferation. Thus, polymeric nanofibers made of biocompatible and biodegradable polymers attracted remarkable attention due to their potential use in tissue engineering as temporary templates for cell seeding, migration, proliferation, and differentiation [2,6,7,8,9,10]. The mats can also serve in advanced drug delivery systems [5,8] and many other applications [1,5,6,11].

Electrospinning is fast developing along two directions to increase its capability of treating polymers. One is the development of multifluid processes such as coaxial [12] and modified coaxial [13], triaxial [14] and modified triaxial [15], side-by-side [16], and other complicated processes [17]. These processes improve the capability of electrospinning in creating complex nanostructures such as core-shell [18], Janus [19], tri-layer core-shell [20], and other complicated structures [21]. The other is the combination of electrospinning with other material treatment methods to endow the electrospun nanofibers improved properties or functional performances [22].

Different natural and synthetic polymers have been used as manufacturing materials for the electrospinning of nanofibers [1,5,6]. Synthetic biodegradable polymers offer easier processability for electrospinning and more controllable nanofibrous morphology than natural polymers [23]. The reason is that manufacturing of synthetic polymers is reproducible in providing similar molecular weight and polydispersity whereas these parameters may differ in each batch of natural polymers [24] and it significantly affects the properties of the solution for electrospinning. A frequently electrospun synthetic biodegradable polymer is polycaprolactone (PCL) [1,6,10,25]. It possesses good mechanical properties and long-term stability from a few months up to 3 years in vivo [26,27]. However, its hydrophobic nature limits the usage in a predominantly hydrophilic bio-environment of drug delivery and tissue engineering applications. Therefore, its modification either by controlling surface potential [28], or with hydrophilic synthetic and biopolymers opens new horizons for bioapplications [29,30,31]. The modifications can be performed either by grafting hydrophilic moieties onto PCL [32,33,34,35] or by the use of amphiphilic blends, e.g., with gelatin or poly(ethylene oxide) (PEO) [30,31,32,33,34,35,36,37,38,39,40]. 

The PEO is well-known to suppress protein adhesion [41] and thus could be applied as an anti-inflammatory polymeric coating for implantable biomaterials and devices [42]. Both PCL and PEO are extensively studied polymers for electrospinning due to their biocompatibility and biodegradability [43,44,45,46]. Several publications utilized PCL and PEO polymers as amphiphilic nanofiber substrates [31,35,47,48,49,50]. The studies have shown auspicious properties, such as very good biocompatibility [36], tunable drug release profiles [29], and the differentiation of mesenchymal stem cells on the nanofiber substrates [50]. 

A simple and efficient method for the modification of nanofibrous mats is their plasma processing. The nanofibers can be coated by plasma polymers containing desired functional groups such as carboxyls, anhydrides or amines without affecting their bulk properties [51,52,53]. Such plasma polymer coatings make the PCL mats less hydrophobic [54], and the functional groups can be beneficially used to attach drugs and immobilize proteins [53,55]. 

The PCL/PEO nanofibers are routinely processed from their solution in toxic solvents such as chloroform, dimethylformamide, tetrahydrofuran or 1,1,1,3,3,3-hexafluoro-2-propanol [56,57,58]. Although no harmful effects have been observed, these chemicals should be preferentially replaced by less or rather non-toxic solvents [59]. In this work, we processed nanofibers from PCL, PEO and their blends in different ratios using acetic and formic acids solvents. The mats were electrospun from wired electrode because this type of technology has a much higher production yield compared to commonly employed syringe electrospinning processes. The PCL/PEO nanofibers exhibited filamentary sub-structures, but the segregation of the immiscible PCL and PEO polymers into larger domains was excluded by the material characterization. It was concluded that PCL and PEO created mixed interpenetrating networks at the nanometer scale and the synergy of the PCL and PEO blend led to a significantly increased Young’s modulus compared to pure PCL or PEO mats. The bioactive and hydrophilic amine plasma polymer coating on the PCL/PEO mats modified the hydrolytic stability and the mechanical properties. Thus, the PCL/PEO electrospinning and plasma processing of nanofibers can be combined to tune the functional properties of nanofibrous mats.

## 2. Materials and Methods

Chemicals. Acetic acid (99%, p.a. grade) and formic acid (98%, p.a. grade) were purchased from Penta s.r.o. (Praha, Czech Republic). PCL flakes with *M*_n_ = 80,000 g·mol^−1^, PEO with *M*_n_ = 100,000 g·mol^−1^, cyclopropylamine (CPA, 98%) and 4-trifluoromethyl benzaldehyde (TFBA, 98%) were purchased from Sigma-Aldrich (Steinheim am Albuch, Germany) and used as received. Argon (99.998%) was supplied by Messer Technogas s.r.o. (Praha, Czech Republic), ultrapure water (UPW) of type I (according to ISO 3696) was prepared on Direct Q3 UV Water Purification System (Merck Millipore, MA, USA).

Electrospinning of nanofibers. The PCL, PEO and PCL/PEO mixed electrospun nanofibers were prepared by electrospinning the 9 wt.% and 11 wt.% polymer solutions dissolved in a mixture of acetic acid (2 weight parts) and formic acid (1 weight part). Different PCL:PEO weight ratios (100:0, 75:25, 50:50, 25:75, 85:15, 90:10, 5:95 and 0:100) solutions were stirred (300 rpm) for 24 h at room temperature (21–25 °C) and electrospun using the Nanospider™ NSLAB 500 (ELMARCO, Praha, Czech Republic). The electrospinning was carried out with a 20 cm long wired electrode at the voltage 40–55 kV and the interelectrode electrospinning distance 100 mm. The high-voltage electrode rotated at 5 rpm and the fabric collector at the grounded electrode moved at 50 mm/min. The fabrication was performed at room temperature, ranging between 21–25 °C, and humidity ranging between 35–40%. The electrospinning time varied from 60 to 90 min and the solution was changed every 20 min to minimize concentration changes in the solution. The resulting nanofibrous mats were compact and flexible foils.

Coating of nanofibers by amine plasma polymer. The electrospun mats cut into 5 × 5 cm pieces were coated by plasma polymer CPA film (PP-CPA) deposited in low pressure pulsed radio-frequency (13.56 MHz) CPA/Ar plasma (50 Pa, 28 sccm of Ar, 2.8 sccm of CPA, pulse duty cycle 33%, repetition frequency 500 Hz, radio-frequency power 150 W, deposition time 30 min) as described in our previous work [60]. Prior to the deposition, the mats were pretreated by pulsed Ar plasma for 10 min. 

Scanning electron microscopy. The surface of the samples was imaged by SEM Tescan LYRA3 in secondary emission mode (10 kV acceleration voltage, working distance 9 mm). Prior to the imaging, the samples were coated with a 10 nm thick gold film deposited by RF magnetron sputtering in order to avoid charging of the surface. 

X-ray photoelectron spectroscopy. The XPS data for a surface chemical characterization of the pure and amine coated nanofibers were acquired using a spectrometer Axis Supra (Kratos Analytical, England). The maximum lateral dimension of the analyzed area was 0.7 mm. To avoid differential charging of samples, spectra were acquired with charge neutralization in overcompensated mode. The spectra were subsequently normalized by shifting aliphatic carbon component to 285.0 eV. The pass energy of 20 eV was used to attain quantitative composition and well-resolved spectra for fitting. The fitting of XPS C1s spectra with individual components was performed in the CasaXPS software (version 2.3.17) after the subtraction of the Shirley-type background employing Gaussian–Lorentzian (G-L) peaks with the fixed G-L percentage 30% (GL30). Constrains of the full width at a half maximum (FWHM) and the peak positions were applied for the fitting of pure PCL and PEO C1s; reported in Supporting Information Figure S1. The following components of the carbon chemical environment were assumed: aliphatic carbon, CH_x_, at 285.0 eV (for PCL and PEO), carbon singly bonded to oxygen, C-O, at 286.4 eV (for PCL and PEO), carbon double bonded to oxygen, C = O and O-C-O, at 288.0 eV (for PEO only), and carbon of carboxyl/ester group, COOR, at 289.0 eV (for PCL and PEO). The model for the fitting of PCL/PEO C1s used three components: PCL with the line shape extracted from the spectra of pure PCL mat, PEO with the line shape extracted from the spectra of pure PEO mat, and the component attributed to aliphatic carbon contamination (standard GL30 line shape at 285.0 eV, FWHM free but consistently around 1 eV). 

The quantification of nucleophilic groups on the surface-coated nanofibers was made by surface derivatization utilizing TFBA [61,62]. Due to the high sensitivity of TFBA to moisture and oxygen, the reaction with TFBA had to be carried out in a dry Ar atmosphere. The examined CPA-coated sample was put on the top of the glass bead placed inside the 100 mL flask. Then, 0.1 mL of TFBA was dropped carefully to the flask in such a way to avoid the contact of liquid with the sample surface. Then, the flask was closed and the reaction between CPA covered nanofibers and evaporated TFBA was allowed to proceed for 90 min. The density of primary amines [NH2] groups, expressed in at.%, was then calculated from the fluorine and carbon atomic content measured by XPS [63,64]. 

Differential scanning calorimetry. The DSC was performed using the DSC Discovery (TA Instruments). Each sample (5–10 mg) was firstly cooled to −85 °C. The first heating was employed from −85 to 150 °C at the rate 10 °C⋅min^−1^. The temperature of 150 °C was maintained for 5 min to destroy any thermal history, the sample was cooled to −80 °C at 10 °C⋅min^−1^ (the first cooling), and then heated to 150 °C at 10 °C⋅min^−1^ (the second heating). Raw data were processed using the TRIOS software to obtain the melting temperature ($T_{m}$) and heat of fusion ($\Delta H_{m}$). The crystallinity (*α*_c_) was calculated from the first DSC heating run according to

(1) $$\alpha_{c}\left(\%\right)=\left(w_{PCL}\cdot \Delta H_{m}+w_{PEO}\cdot \Delta H_{m}\right)/\left(w_{PCL}\cdot \Delta H_{m,\;PCL}^{0}+w_{PEO}\cdot \Delta H_{m,\;PEO}^{0}\right)$$

where $w_{PCL}$ and $w_{PEO}$ are the PCL and PEO weight fractions in the sample, respectively; $\Delta H_{m}\;$ is heat of fusion of the nanofiber sample; $\Delta H_{m,PCL}^{0}$ is heat of fusion of 100% crystalline PCL (135.44 J·g^−1^) [65]; and $\Delta H_{m,PEO}^{0}$ is the heat of fusion of 100% crystalline PEO (196.80 J·g^−1^) [66].

Hydrolytic degradation tests. The stability in water was investigated by hydrolytic degradation tests carried out in an incubator at 37 °C in ultrapure water (UPW). Nanofibrous mats were cut into 1 × 1 cm pieces, immersed in UPW and removed at the given time from vials with UPW. Finally, the mats were dried at 30 °C in vacuo until the constant mass was reached. Mass loss was calculated according to the formula

(2) $$\mathrm{Mass}\;\mathrm{loss}\;(\%)=(w_{0}-w_{t})/w_{0}\times 100$$

where $w_{0}$ is the mass of the sample, and $w_{t}$ is the mass of the dried sample. Each measurement was an average of 3 specimens and data were expressed as mean ± standard deviation.

Tensile tests. Tensile tests were performed employing the tensile tester (Zwick Roell Z010, Ulm, Germany) at laboratory temperature. Specimens were stripes with dimensions of 5 × 30 mm (width × length). Their thickness was measured with a micrometer screw, similarly to our previous work [62] that proved the sufficient relative comparability of such measurements. A 500-N load cell was used for the measurement, with the cross-head speed of 5 mm·min^−1^ corresponding to the 50%·min^−1^ deformation rate. The gauge length was 10 mm and the 0.05-N preload was used for all the samples. Ten stripes were measured from each sample and data were averaged to get the standard deviation.

## 3. Results and Discussion

It was reported for PEO electrospinning that the key factors influencing the formation of the beaded fibers are the viscoelasticity of the solution, charge density carried by the jet, and the surface tension of the solution [67]. Defects in the form of beads were commonly observed in the electrospinning of other polymers if the polymer concentration was below or above the optimum value [68,69]. Previously, we investigated the PCL electrospinning with Nanospider™ Elmarco and concluded that the best nanofibrous structures were obtained for the polymer concentration of 9 wt.% and the applied voltage of 50–55 kV [25]. In this work, we investigated by SEM the morphology of the mats electrospun from PCL/PEO blends at various polymer concentrations and voltages. The best structured nanofibers were obtained at 9 wt.% and 55 kV for the PCL/PEO mixture containing 50–100 wt.% of PCL (samples denoted as PCL, PCL75/PEO25 and PCL50/PEO50), whereas 11 wt.% and 40 kV appeared as optimum for the PCL concentrations below 50 wt.% (denoted as PCL25/PEO75, PCL15/PEO85, PCL10/PEO90, PCL5/PEO95 and PEO).

As reported in our previous paper [25], the average diameter of PCL electrospun fibers was (150 ± 50) nm for 40 kV and 7 wt.% of PCL in the solution and it increased for higher concentrations (9—12 wt.%), whereas the voltage did not influence the diameter within the range of statistical errors. A similar conclusion was drawn from a more complete study of the PCL fiber diameters shown in Figure S2 of Supporting Information, but the variance of the fiber diameters does not prove the statistical significance between 9 and 11 wt.%. We tested different PCL:PEO mixtures only for 9 and 11 wt.% and we did not observe any systematic variation of the mean fiber diameter for varied PCL:PEO ratio. The diameter was between 250 and 450 nm.

**Figure 1:**
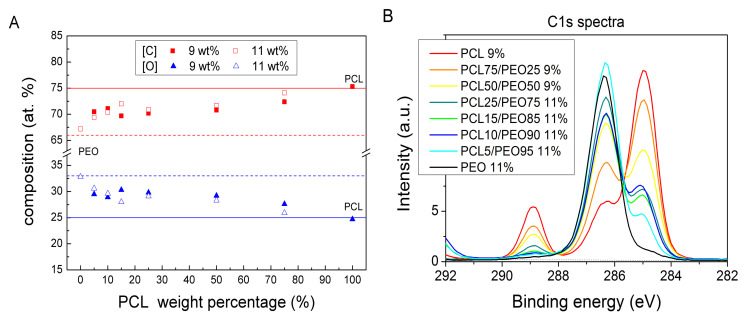

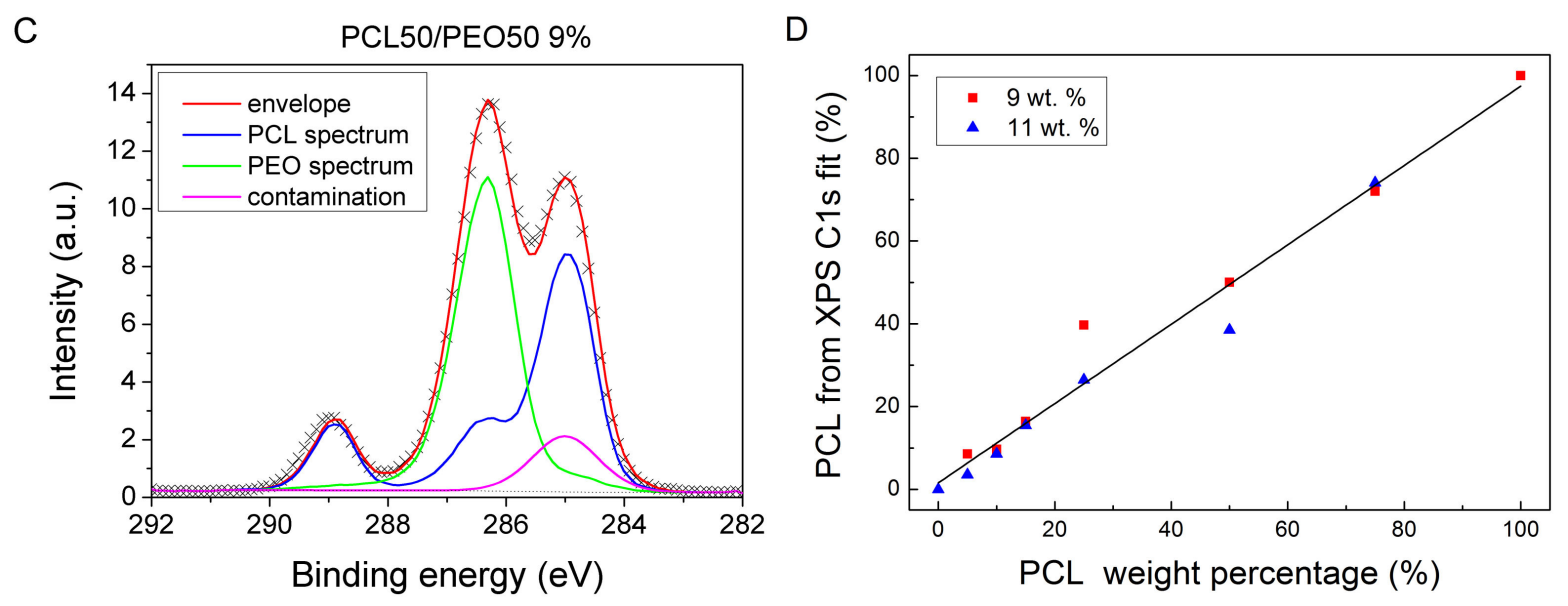
Analyses of PCL/PEO nanofibrous mats by XPS: (**A**) elemental composition in dependence on the PCL weight percentage, (**B**) high-resolution C1s XPS spectra of PCL, PEO and PCL/PEO mats, (**C**) example of C1s fitting using the model based on pure PCL, PEO and aliphatic carbon contamination components, and (**D**) linear relation between the PCL concentration in the mats (obtained by fitting C1s data) and the PCL weight percentage in the electrospinning PCL/PEO mixture.

Theoretical elemental composition of pure PCL and PEO are 75 at.% of carbon, 25 at.% of oxygen, and 66 at.% of carbon, 33 at.% of oxygen, respectively. For the investigated samples, the experimentally obtained elemental composition agreed well with the theoretical predictions (Figure 1A). The trend in the elemental composition of the mixed PCL/PEO nanofibers reflected the varied composition of the electrospinning solution. Figure 1B shows a gradual change in the C1s spectra from PCL to PEO with the blend ratio. The C1s signal offers higher sensitivity for the assessment of the PCL/PEO ratio in the surface of nanofibers. Therefore, the high-resolution C1s XPS signal of PCL and PEO mats was fitted by appropriate carbon chemical environments (Supporting Information Figure S1) and these fits served as the basis for the analysis of PCL/PEO blended mats. The pure PEO contains mainly peak corresponding to carbon single bonded to oxygen (C-O), whereas PCL also contains characteristic aliphatic and ester peaks not present in PEO. It provided the framework for the fitting of the C1s signal of PCL/PEO mats by pure PCL and PEO components as shown in Figure 1C. Additionally, a component corresponding to a contamination by aliphatic carbon was required to achieve a good fit. This model allowed us to calculate the PCL percentage in the surface layer of electrospun nanofibers (Figure 1D). The linear dependence shown in Figure 1D revealed that the top surface layer (approx. 5 nm estimated from the XPS information depth, i.e., thickness from which a majority of the detected signal originates) had the same PCL/PEO composition as the PCL/PEO blend used for the electrospinning. 

**Figure 2:**
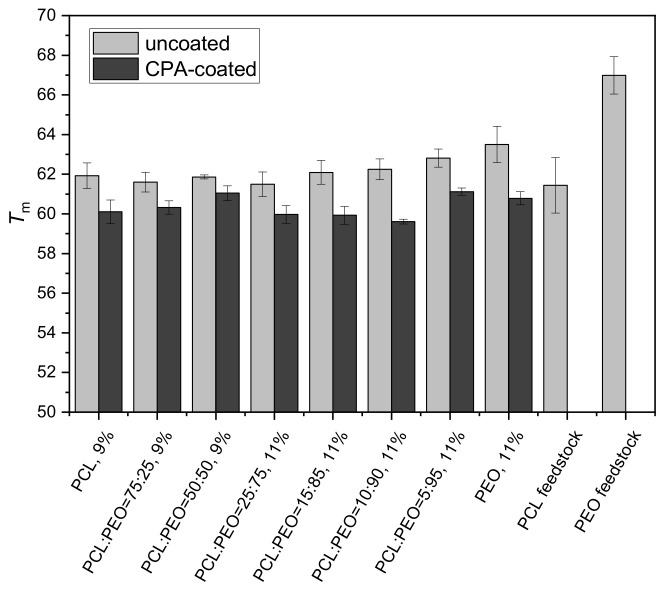
Melting temperature (*T*_m_) obtained from the DSC thermographs of uncoated and CPA-coated PCL/PEO electrospun mats during the first heating cycle. The PCL/PEO ratio and polymer weight concentration in the electrospinning solution are given on the axis.

**Figure 3:**
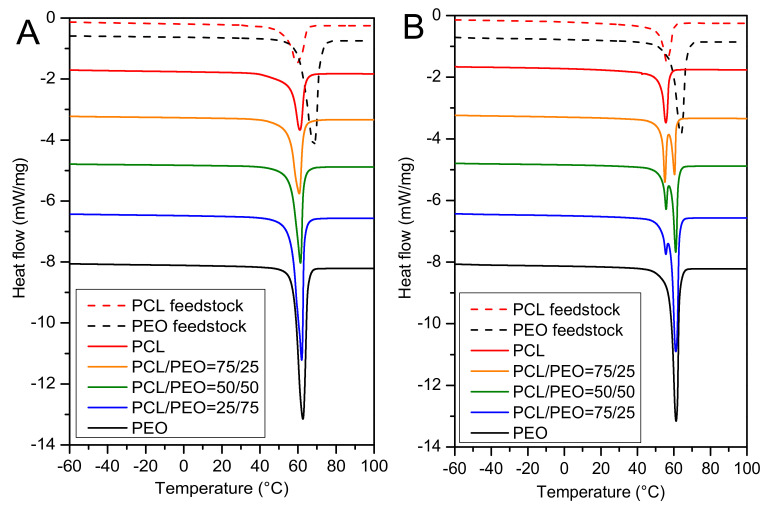
DSC thermographs of (**A**) the first heating cycle showing a single melting peak, and (**B**) the second heating cycle revealing two melting peaks demonstrating two separate polymers in the mixed samples. Only the as-prepared PCL, PCL75/PEO25, PCL50/PEO50, PCL25/PEO75 and PEO samples are shown for a better clarity of the graphs. The thermographs of all the samples can be found in Supplementary Information Figure S3.

The DSC proved the semicrystalline nature of the nanofibers. The crystallinity of the PCL electrospun nanofibers, (67 ± 4)%, was by 7% higher than for the neat PCL feedstock (Table S1) but their melting point did not differ within the range of standard deviations (Figure 2). The PEO nanofibers and the PEO feedstock had a different crystallinity ((81 ± 3)% and (94 ± 5)%, respectively), and the PEO mat exhibited a lower melting temperature than the PEO feedstock ascribed to a less developed crystalline order. The first and the second DSC heating (Figure 3A,B, respectively) revealed the striking difference between the as-prepared electrospun PCL/PEO mats and the mats processed to melt during the first DSC heating. The second heating demonstrated two melting peaks attributed to the separate melting of PCL and PEO crystalline domains. Thus, we can conclude that the as-prepared electrospun mats from the PCL/PEO mixtures are not composed of separate PCL or PEO microscopic domains, but each nanofiber consists of a PCL and PEO blend. Previously, the investigation of casted PCL/PEO films led to the conclusion that these two semicrystalline polymers are immiscible at room temperature [70]. In the electrospun nanofibers, the limited radial dimension of the nanofibers may restrict the length scale of phase separation between the immiscible constituents, and the separated domains cannot be as large as the few micrometers observed in the cast films of immiscible polymers [71].

The electrospun nanofibrous mats were coated with plasma polymerized cyclopropylamine (PP-CPA) film, as described in our previous work [51,60] and Section 2 (Materials and Methods) above. It added amine functionality to the inert nanofibrous surface. The XPS analyses confirmed that the atomic composition of the PP-CPA was 81, 5, and 14 at.% of carbon, oxygen, and nitrogen, respectively, for all the coated mats. Thus, the nature of polymer nanofibers did not play any role for the deposition of the amine film. It was not possible to quantify the density of primary amine groups NH_2_ directly from the high resolution C1s or N1s curve fitting due to very small differences in the binding energies (BE) of primary amines and other nitrogen chemical environments present in the PP-CPA [60]. To confirm and quantify the primary amines in the PP-CPA films on the surface of nanofibers, the derivatization of hydrophilic groups was performed using TFBA [61,63,64]. The concentration of primary amine groups on CPA-coated electrospun mats, as determined by XPS, was 1.5 at.%. Besides primary amines, there are other reactive functional groups and radicals in the amine plasma polymer films [55] that can attach proteins and other compounds.

The thickness of the PP-CPA film on the top mat surface was 216 ± 8 nm. We have shown previously that the plasma polymerization can penetrate quite deep into the nanofibrous mats, but the film thickness and its composition change with the penetration depth [62]. In this work, the deep penetration of the plasma processing into the nanofibrous structure was confirmed by a change of the melting temperature obtained from the DSC, because *T*_m_ is the bulk property rather than the property of the top mat surface. All the samples of the nanofibrous mats coated with the PP-CPA film possessed a lower *T*_m_ (Figure 2) compared to the uncoated ones, signifying that the PP-CPA film deposition preceded by the Ar plasma treatment partially changed the structure of the material.

**Figure 4:**
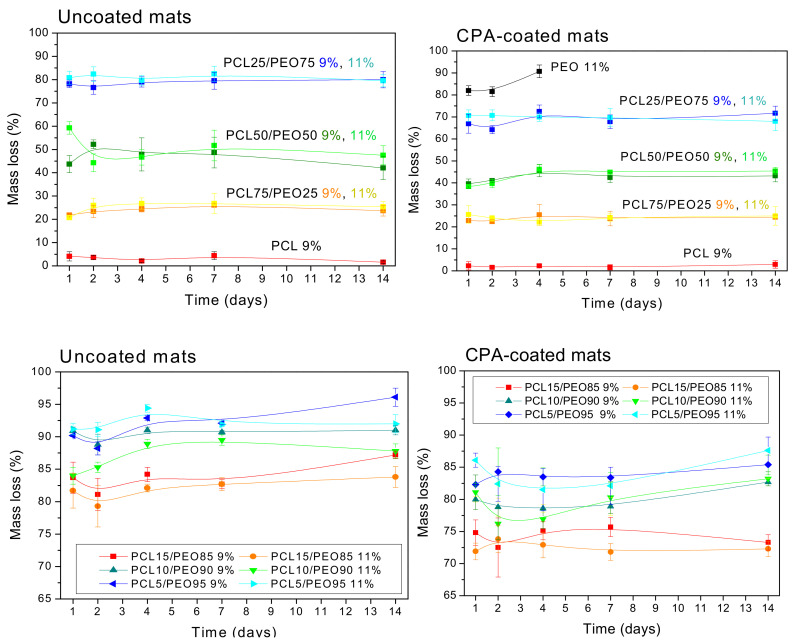
Hydrolytic degradation of the as-prepared (uncoated) and the CPA-coated nanofibrous mats in the period of 14 days. Top figures shows overview of changes with different PCL/PEO ratios and the bottom figures provide details in the PCL/PEO.

The hydrolytic stability of the uncoated mats followed their composition, i.e., the percentage of hydrophilic PEO and hydrophobic PCL (Figure 4, left part). The PEO polymer easily dissolves in water due to its hydrophilic nature, whereas the hydrophobic PCL is stable in water without a significant mass loss. Since the PEO nanofibrous mats dissolved immediately in water, their results are not presented in Figure 4. Accordingly, the mats made from the large PEO content mixtures, 90–95%, exhibited a high mass loss in the range of 85–95%. The PCL mats, on the other hand, lost only around 4% of their initial mass, and the PCL50/PEO50 mats lost approximately 50% during the 14 days of testing. Small deviations from the average mass loss observed for the two PCL50/PEO50 samples electrospun from the polymer solutions of two different concentrations, 9 and 11 wt.%, are attributed to material inhomogeneities. In conclusion, changing the PCL/PEO mixture composition tunes continuously dissolution of the PCL/PEO electrospun mats, and the mass loss was slightly lower than expected from the PCL and PEO concentrations in the electrospinning mixture.

**Figure 5:**
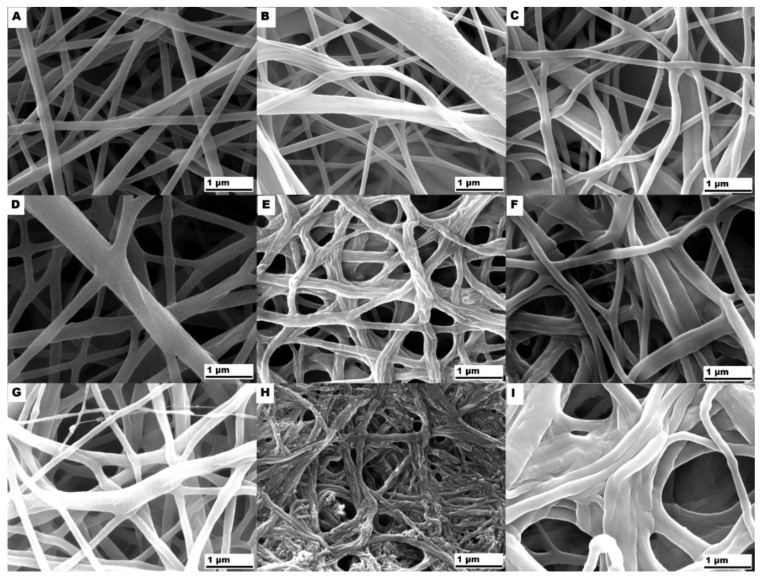
SEM micrographs showing the morphology of nanofibrous mats undergoing hydrolytic stability tests: (**A**) pure PCL, (**B**) pure PCL after 14 day of immersion in UPW, (**C**) CPA-coated PCL after 14 day of immersion in UPW, (**D**) PCL50/PEO50, (**E**) PCL50/PEO50 after 14 day of immersion in UPW, (**F**) CPA-coated PCL50/PEO50 after 14 day of immersion in UPW, (**G**) PCL15/PEO85, (**H**) PCL15/PEO85 after 14 day of immersion in UPW, (**I**) CPA-coated PCL15/PEO85 after 14 day of immersion in UPW. The polymer concentration in the electrospinning solution was 11 wt.% for PCL15/PEO85 and 9 wt.% for PCL and PCL50/PEO50.

The PP-CPA coating of nanofibers partially changed their bulk properties in terms of the structure (lowering the melting temperature, Figure 2), and the coating partially protected the mats against dissolution in water, as demonstrated by the mass loss in the right section of Figure 4. The PEO mats did not completely dissolve after the first contact with water, and a thin layer of the sample remained stable for four days. The nanofibrous mats with significant PEO content (e.g., PCL50/PEO50, PCL25/PEO75, and higher) also exhibited better stability in water. Figure 5F,I demonstrated that the nanofibers are uniformly coated by the plasma polymerization of CPA, and the difference in the mass loss evidenced that the coating penetrated deeper in the porous mat structure, as already discussed above.

The SEM of partially dissolved mats for significant PEO content (Figure 5E,H for PCL50/PEO50 and PCL15/PEO85, respectively) revealed the morphology of isolated filaments forming the nanofibers. Since they remained after etching in water, they had to be composed of mainly PCL. It was reported for the PEO/polystyrene electrospun nanofibers that the minority PEO component (below 20 wt.%) in the immiscible PEO/polystyrene blend formed short isolated fibrils due to the extensional flow encountered during fiber spinning [72]. Elongated porous structures were also observed in the PS/PEO/PCL electrospun nanofibers etched in water, and the existence of two melting temperatures confirmed the phase segregation of PCL and PEO [71]. From this point of view, it is interesting to recall our DSC results that did not show two melting temperatures of the PCL/PEO nanofibers. The filamentary morphology of our PCL/PEO nanofibers after etching in water revealed certain phase segregation. Unlike in [71], the DSC of our PCL/PEO nanofibers did not show two melting peaks in the first heating cycle (Figure 3A), i.e., we did not confirm the phase segregation of PCL and PEO. We attribute it to the much thinner electrospun fibers (250–450 nm as measured from SEM micrographs) than those studied by Samanta et al. [71], and conclude that the DSC cannot provide information about the nano-sized domains of separated PCL and PEO. The immiscibility of PCL and PEO in melted PCL/PEO blends was, however, confirmed by the DSC in the second heating cycle that revealed two melting temperatures (Figure 3B). 

**Figure 6:**
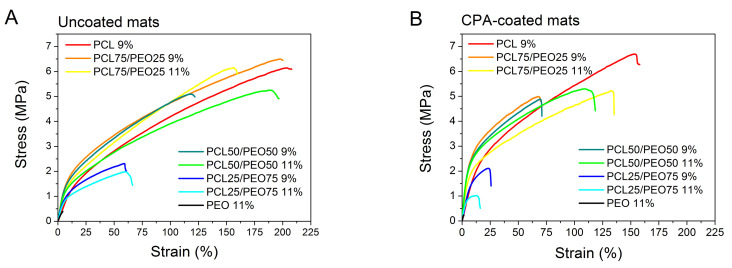

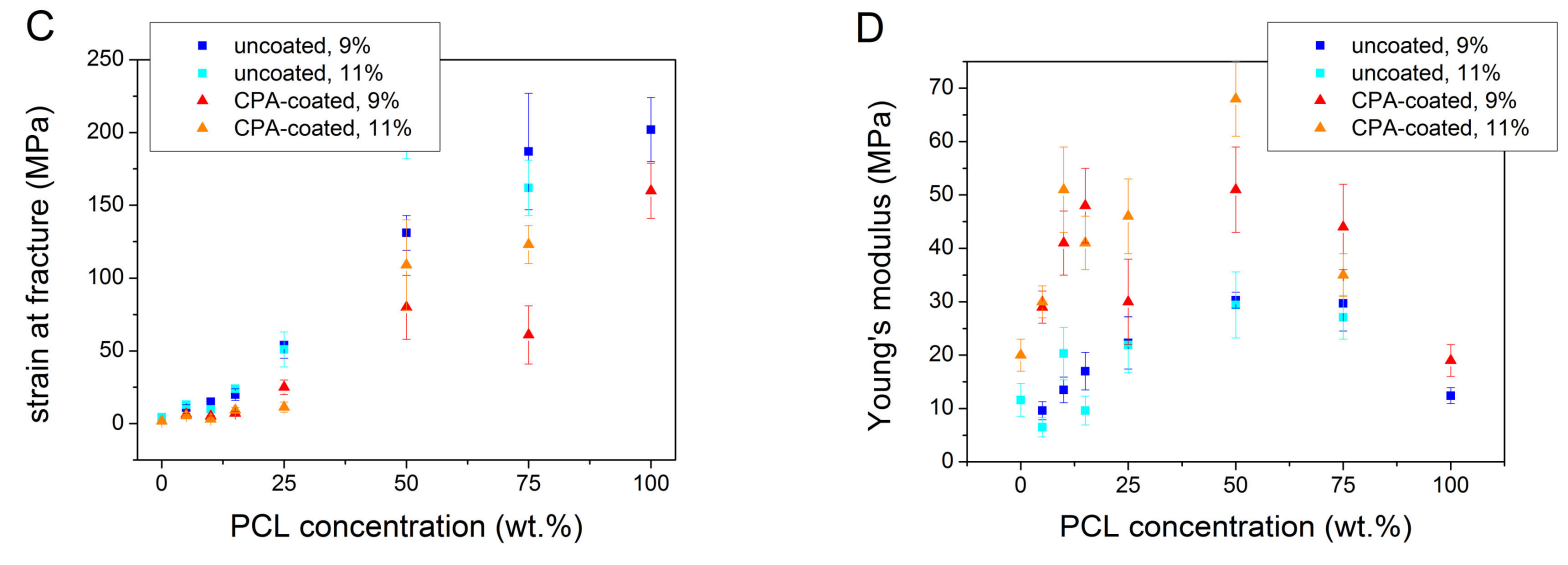
Stress–strain curves showing tensile properties of (**A**) uncoated and (**B**) CPA-coated nanofibrous mats. The polymer concentration (wt.%) and the composition of the polymer mixture in the electrospinning solution are given in the figure captions. Stress–strain curves for high concentration PEO mixtures are shown in the Supporting Information Figure S4. Graphs (**C**) and (**D**) show dependencies of the mechanical properties calculated from the stress–strain curves on the weight concentration of PCL.

The mats electrospun from the mixtures with high PCL concentrations were more resistant to deformation, and their fracture strain was much higher, as demonstrated by the stress–strain curves in Figure 6A and the strain values at the fracture (Figure 6C). An exciting phenomenon of increased Young’s modulus was observed for the blended PCL/PEO nanofibrous mats. The pure PCL and PEO mats possessed a Young’s modulus of about 12 MPa, but the mats made of the PCL75/PEO25 and PCL50/PEO50 mixtures exhibited 2.5-fold higher stiffness (Figure 6D). It can be attributed to the synergistic effect of the PCL/PEO mixing at the nanoscale. Thus, the mechanical tests are in agreement with the conclusions drawn from the DSC measurements. It is worth noting that the samples PCL75/PEO25 (9 wt.%) and PCL50/PEO50 (11 wt.%) with a higher Young’s modulus had a similar strength and ductility to the pure PCL mats. Therefore, they can be used if a higher Young’s modulus is desired and the solubility in water needs to be tuned (Figure 4) for the envisaged applications, such as for wound healing and drug delivery.

The plasma processing of mats by Ar pretreatment and PP-CPA coating significantly enhanced the mat stiffness for 5–50 wt.% of PCL in the PCL/PEO blend (Figure 6B,D). Contrarily, a decrease was observed in the stress and strain at fracture. These trends can be attributed to an increased polymer cross-linking that increases the polymer stiffness but decreases its ductility [73]. Indeed, the plasma–polymer interaction involves the effect of ions and UV radiation that deliver the necessary energy for polymer cross-linking [74]. 

**Figure 7:**
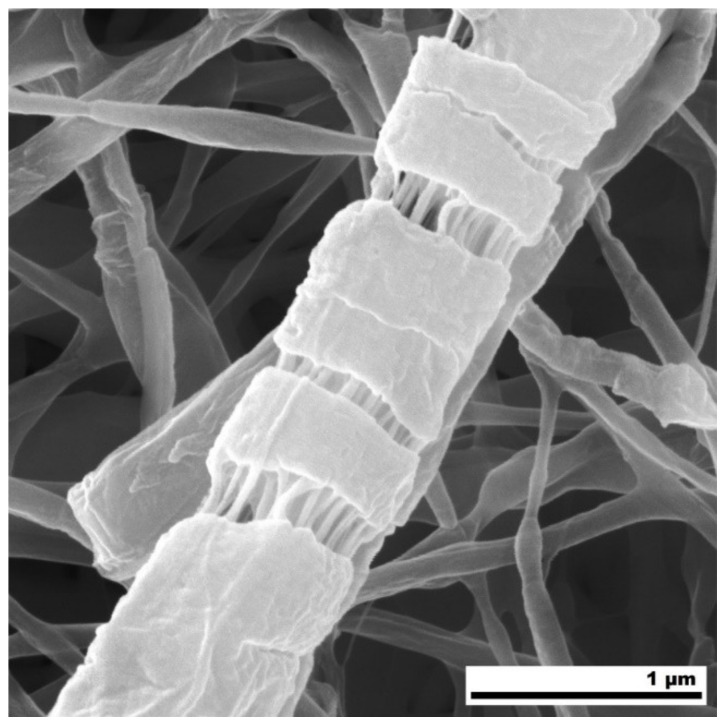
SEM micrograph of PCL15/PEO85 nanofibrous mats after the tensile test leading to the material fracture. It revealed an internal structure of nanofibers consisting of PCL-based fibrils (supposed to be PCL/PEO blend) in the fragile PEO matrix.

The SEM investigation of the nanofibrous mats undergoing the fracture during the tensile tests confirmed a remarkable filamentary sub-structure of the PCL/PEO nanofibers that was proposed above on the basis of water etching of high PEO mats (Figure 5). The filamentary structure after the tensile tests was observed for nanofibers with the PCL content 15 wt.%, as demonstrated by the high-resolution SEM micrograph of the fractured PCL15/PEO85 mat in Figure 7. It was confirmed for both the uncoated and CPA-coated mats (further micrographs are in Supporting Information Figure S5). Based on the differences in the mechanical properties of PCL and PEO, we conclude that the filaments are PCL-based (either pure PCL or high PCL concentration PCL/PEO blend) in the fragile matrix composed mainly of PEO. This conclusion is in agreement with the water etching results. The DSC did not confirm the phase segregation of PCL and PEO, because only one melting temperature was observed in the first heating cycle. It is attributed to nano-sized separated PCL filaments inside the PEO matrix that represent the well-blended PCL/PEO mixture from the microscopic point of view. The XPS C1s analysis (Figure 1D) confirmed that the top surface (approx. 5 nm) of PCL/PEO nanofibers was composed of the PCL/PEO blend with the same composition as the electrospinning solution. Therefore, the near subsurface area of the nanofibers also contained some PCL filaments. Thus, the characterization methods led to the conclusion that PCL and PEO created mixed interpenetrating networks at the nanometer scale.

## 4. Conclusions

The PCL/PEO blend nanofibers with varied PCL:PEO ratios from 5:95 to 75:25 were successfully processed into the electrospun mats and further coated by amine plasma polymer that can provide increased hydrophilicity and surface functionality for the attachment of additional compounds. The filamentary structure of each nanofiber was disclosed by SEM after hydrolytic degradation, and tensile tests carried out on the PCL/PEO mats with a high concentration of PEO. Based on the XPS and DSC, we concluded that the filamentary sub-structures of segregated PCL and PEO are organized at the nanometer scale. The DSC also proved the semicrystalline nature of the nanofibers.

The varied PCL:PEO concentrations in the polymer solutions tuned continuously dissolution of the PCL/PEO electrospun mats. The degradation rate of PCL/PEO nanofibers was increased compared to pure PCL, and the mass loss was slightly lower than expected from the PCL and PEO concentrations in the electrospinning mixture. An exciting synergistic phenomenon of increased stiffness appeared for the high concentration of PCL in the PCL/PEO nanofibrous mats. The pure PCL and PEO mats had a Young’s modulus of about 12 MPa, but the mats made of high concentration PCL in PCL/PEO solution exhibited 2.5-fold higher values. The increase in the PEO content led to up to a 20-fold decrease in the nanofibers’ ductility. The amine plasma polymer coating significantly increased the water stability of the nanofibers in water. The plasma processing of mats significantly enhanced the mat stiffness for 5–50 wt.% of PCL in the PCL/PEO blend. Contrarily, a decrease was observed in the stress and strain at fracture. These trends can be attributed to an increased polymer cross-linking that increases the polymer stiffness, but decreases its ductility.

In conclusion, the synergy effects in the mats composed of PCL, PEO and amine plasma polymer coating enable us to tune the surface functionality, the hydrolytic stability, and the mechanical properties of the biodegradable nanofibrous mats. Such materials can be utilized for applications in wound healing and tissue engineering, or as models in accelerated drug and cosmetics testing. An adjustable degradation rate of PCL/PEO, together with improved mechanical properties expected for a given PCL/PEO ratio, can be advantageous for special applications such as smart drug delivery systems or wound healing.

## Data Availability

The datasets generated or analyzed during the current study are available from the corresponding author on reasonable request.

## Supplementary Materials

The following are available online at https://www.mdpi.com/2073-4360/12/6/1403/s1, **Figure S1.** Fitting of high-resolution XPS C1s signal for pure PCL and PEO mats by Gaussian-Lorentzian peaks assigned to different carbon chemical environment (given in the figure caption), **Figure S2.** Average diameter of electrospun PCL nanofibers in dependence on applied voltage for three different concentrations of PCL in solution. Error bars represent standard deviations of the mean diameter, **Figure S3.** DSC thermographs for all PCL/PEO mats: A) and C) show the first heating with a single melting peak of as-prepared (uncoated) and CPA-coated nanofibers, respectively, and B) and D) show the second heating revealing two melting peaks demonstrating two separate polymers in the samples of uncoated and CPA-coated nanofibers, respectively, **Figure S4.** Stress-strain curves showing tensile properties of uncoated and CPA-coated nanofibrous mats electrospun from high concentration PEO mixtures. The polymer concentration (wt.%) and the composition of the PCL/PEO polymer mixture in the electrospinning solution are given in the figure captions, **Figure S5.** SEM micrographs of CPA-coated nanofibrous mats after tensile test showing the character of the breaking area, A) pure PCL 9 wt.%, B) PCL75/PEO25 9 wt.%, C) PCL50/PEO50 11 wt.%, D) PCL10/PEO90 11 wt.%, **Table S1.** Crystallinity obtained from the DSC thermographs during the first heating of the uncoated and PP-CPA coated PCL/PEO electrospun mats.
