# Supplementary material for: Well-Blended PCL/PEO Electrospun Nanofibers with Functional Properties Enhanced by Plasma Processing
Source: Polymers (Basel). 2020 Jun 22;12(6):1403. doi: 10.3390/polym12061403 (PMC7362260; doi:10.3390/polym12061403)
Supplement: Supplementary file 1 [file polymers-12-01403-s001.pdf]

Supporting Information to

Well-blended PCL/PEO Electrospun Nanofibers with Functional Properties Enhanced by Plasma Processing

Authors: Vojtěch Kupka[1,2], Eva Dvořáková[3,4], Anton Manakhov[3,5], Miroslav Michlíček[3,4], Josef Petruš[1,6], **Lucy Vojtová[1] and Lenka Zajíčková[1,3,4*]**

[1] *Central European Institute of Technology - CEITEC, Brno University of Technology, Purkyňova 123, Brno 61200, Czech Republic*

[2] *Regional Centre of Advanced Technologies and Materials and Department of Physical Chemistry, Faculty of Science, Palacký University in Olomouc, 17. listopadu 12,, 779 00 Olomouc, Czech Republic*

[3] *Central European Institute of Technology - CEITEC, Masaryk University, Kamenice 5, Brno 625 00, Czech Republic*

[4] *Department of Physical Electronics, Faculty of Science, Masaryk University, Kotlářská 2, Brno 61137, Czech Republic*

[5] *National University of Science and Technology "MISIS", Leninsky prospect 4, Moscow, 119049, Russian Federation*

[6] *Institute of Materials Chemistry, Faculty of Chemistry, Brno University of Technology, Purkyňova 464/118, 612 00 Brno, Czech Republic*

***Email of corresponding author**: lenkaz@physics.muni.cz

1.1 XPS analysis of PCL, PEO and PCL/PEO mats

The high-resolution C1s XPS signal of PCL and PEO mats was fitted by appropriate carbon chemical environments: aliphatic carbon, **C**H_x, at 285.0 eV (for PCL and PEO), carbon singly bonded to oxygen, **C**-O, at 286.4 eV (for PCL and PEO), carbon double bonded to oxygen, **C**=O and O-**C**-O, at 288.0 eV (for PEO only), and carbon of carboxyl/ester group, **C**OOR, at 289.0 eV (for PCL and PEO). The C1s signal with the fitting is shown in Figure S1.

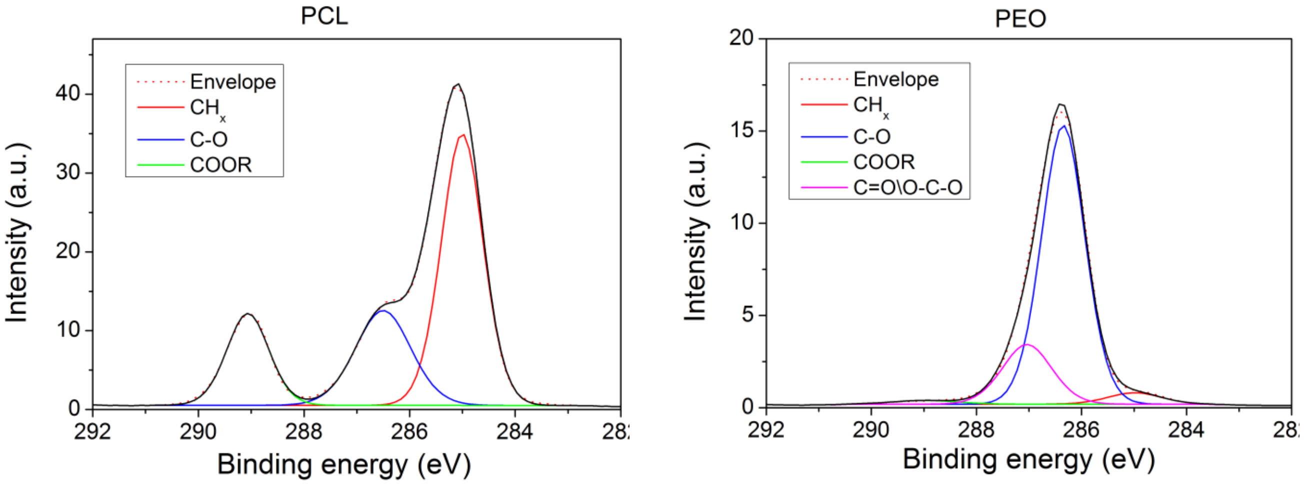

Figure S1. Fitting of high-resolution XPS C1s signal for pure PCL and PEO mats by Gaussian-Lorentzian peaks assigned to different carbon chemical environment (given in the figure caption).

1.2 Average fiber diameter of electrospun PCL

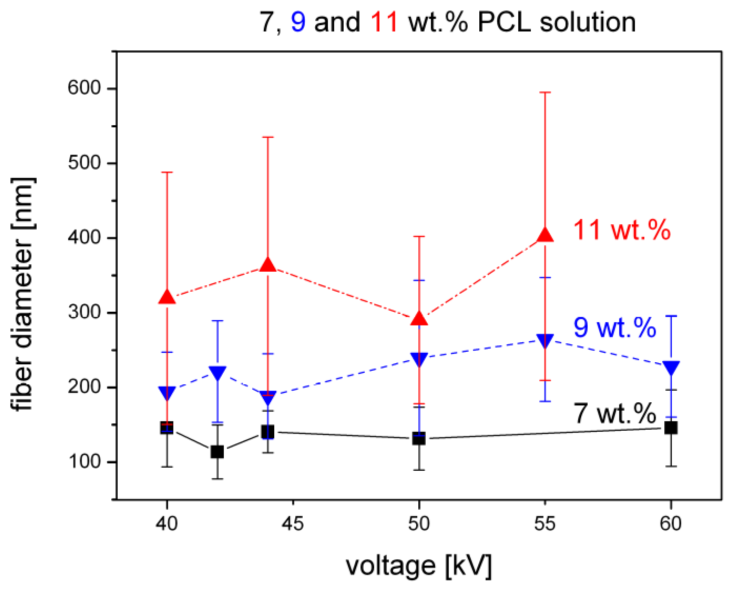

Figure S2. Average diameter of electrospun PCL nanofibers in dependence on applied voltage for three different concentrations of PCL in solution. Error bars represent standard deviations of the mean diameter.

1.3 Structural analysis by differential scanning calorimetry (DSC)

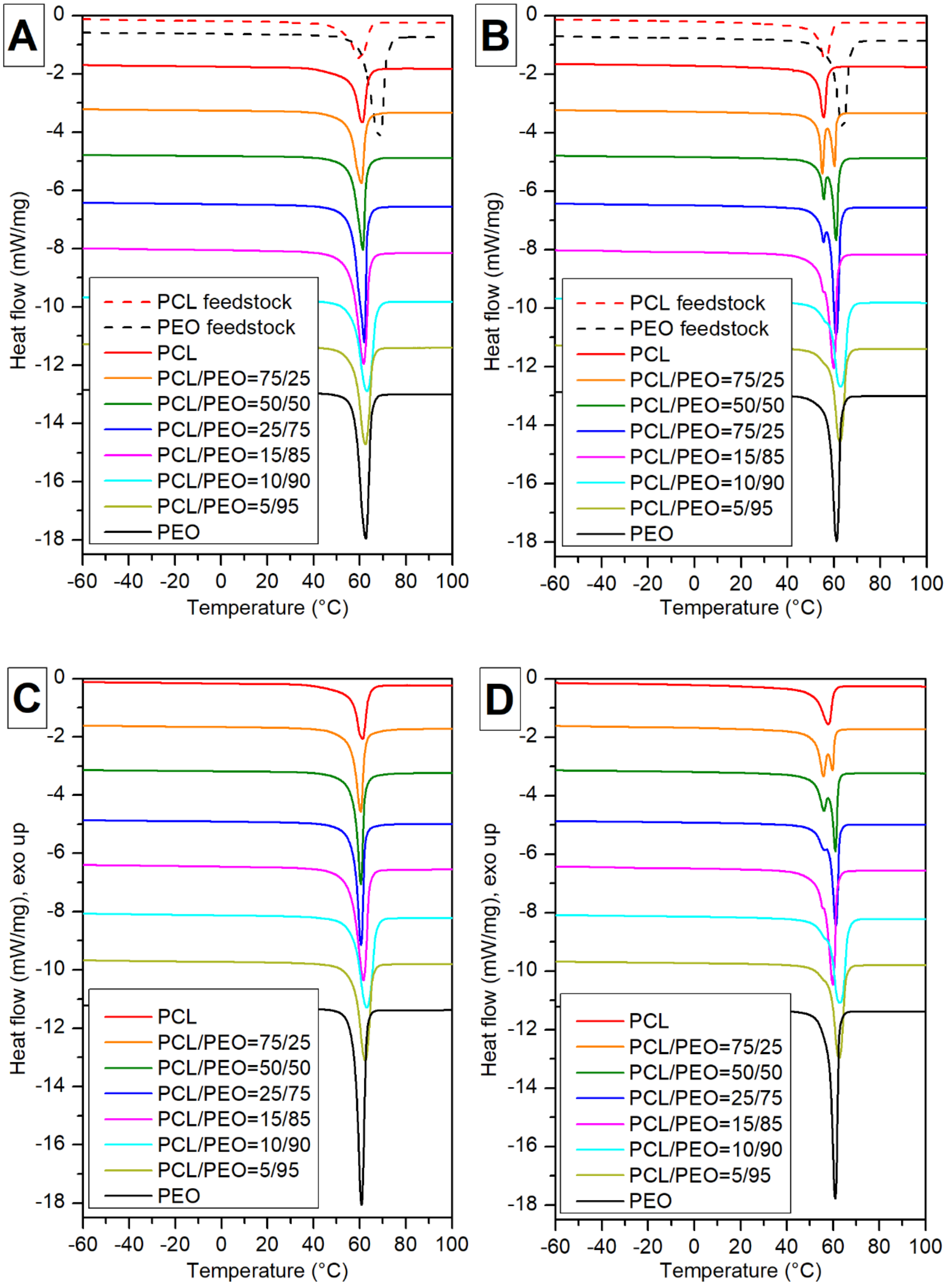

Figure S3. DSC thermographs for all PCL/PEO mats: A) and C) show the first heating with a single melting peak of as-prepared (uncoated) and CPA-coated nanofibers, respectively, and B) and D) show the second heating revealing two melting peaks demonstrating two separate polymers in the samples of uncoated and CPA-coated nanofibers, respectively.

Table S1. Crystallinity obtained from the DSC thermographs during the first heating of the uncoated and PP-CPA coated PCL/PEO electrospun mats.

Sample label	Crystallinity (%)	
	uncoated	PP-CPA coated
PCL, 9%	67 ± 4	62 ± 6
PCL:PEO=75:25, 9%	74 ± 3	66 ± 3
PCL:PEO=50:50, 9%	77 ± 5	76 ± 6
PCL:PEO=25:75, 11%	84 ± 5	77 ± 7
PCL:PEO=15:85, 11%	85 ± 3	82 ± 1
PCL:PEO=10:90, 11%	85 ± 5	84 ± 1
PCL:PEO=5:95, 11%	85 ± 6	85 ± 1
PEO, 11%	84 ± 6	85 ± 2
PCL feedstock	60 ± 4	
PEO feedstock	94 ± 5	

1.4 Tensile test

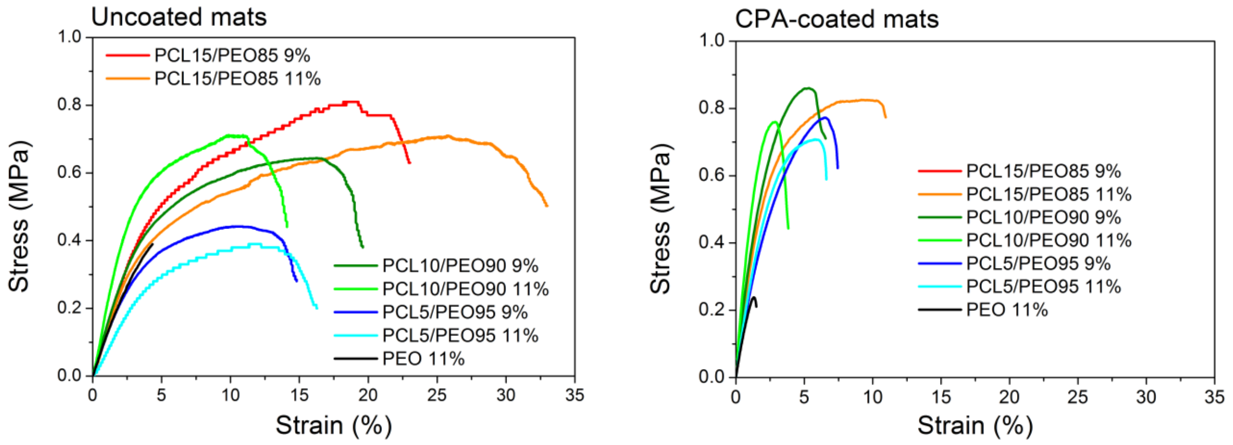

Figure S4. Stress-strain curves showing tensile properties of uncoated and CPA-coated nanofibrous mats electrospun from high concentration PEO mixtures. The polymer concentration (wt.%) and

the composition of the PCL/PEO polymer mixture in the electrospinning solution are given in the figure captions.

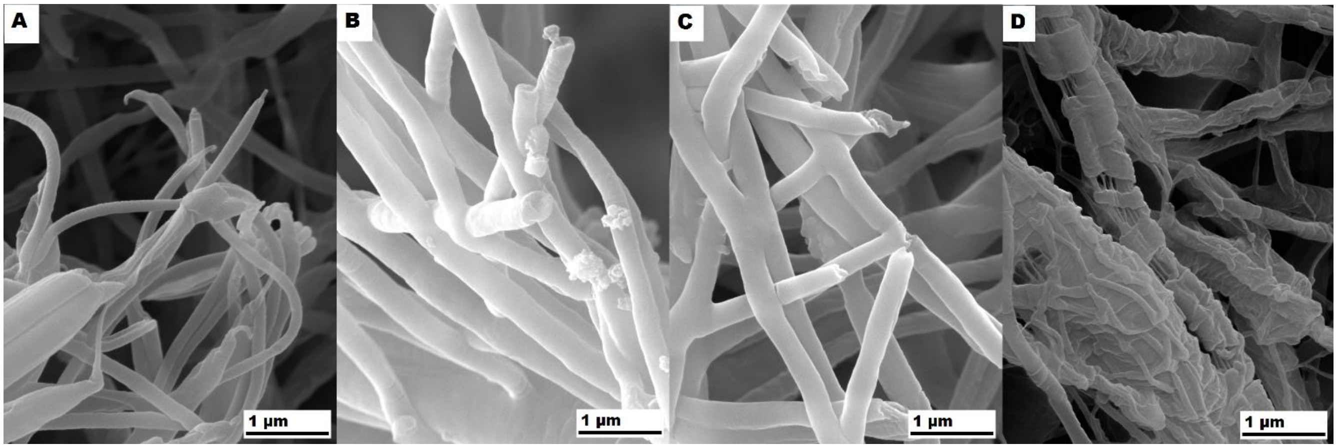

Figure S5. SEM micrographs of CPA-coated nanofibrous mats after tensile test showing the character of the breaking area, A) pure PCL 9 wt.%, B) PCL75/PEO25 9 wt.%, C) PCL50/PEO50 11 wt.%, D) PCL10/PEO90 11 wt.%.
